# Enhanced UV Penetration and Cross‐Linking of Isoporous Block Copolymer and Commercial Ultrafiltration Membranes using Isorefractive Solvent

**DOI:** 10.1002/advs.202403288

**Published:** 2024-07-01

**Authors:** Michael Appold, Sofia Rangou, Sarah Glass, Brigitte Lademann, Volkan Filiz

**Affiliations:** ^1^ Institute of Membrane Research Helmholtz‐Zentrum Hereon Max‐Planck‐Str.1 21502 Geesthacht Germany

**Keywords:** anionic polymerization, block copolymers, foam‐like structure, functionalization, isoporous membrane, self‐assembly, ultrafiltration membrane

## Abstract

Amphiphilic block copolymers are promising candidates for the fabrication of ultrafiltration membranes with an isoporous integral asymmetric structure. The membranes are typically fabricated by the combination of block copolymer self‐assembly and the non‐solvent‐induced phase separation (SNIPS) process resulting in isoporous integral asymmetric membranes. Certainly, all these membranes lack thermal and chemical stability limiting the usage of such materials. Within this study, the fabrication of completely cross‐linked isoporous integral asymmetric block copolymer membranes is demonstrated by UV cross‐linking resulting in chemical and thermal stable ultrafiltration membranes. The UV cross‐linking process of PVBCB‐*b*‐P4VP (poly(4‐vinylbenzocyclobutene)‐*b*‐poly(4vinylpyridine)) block copolymer membranes in dependency of irradiation time, intensity, distance between membrane and UV source and the wavelength is investigated. Furthermore, it is shown that the penetration depths can be increased by soaking the membranes in wave‐guiding solutions before UV cross‐linking is carried out. Moreover, a completely new and easy cross‐linking strategy is developed based on isorefractive solvents resulting in thermal and chemically stable membranes that are cross‐linked through the whole membrane thickness. Finally, the new cross‐linking strategy in isorefractive solutions is transferred to commercial PVDF and PAN‐*co*‐PVC polymer membranes paving the way for more stable and sustainable ultrafiltration membranes.

## Graphical abstract

A membrane in an isorefractive solution is becoming transparent and being penetrated by UV light. The membrane becomes invisible when its refractive index matches that of the solution so that the UV light is able to pass through the membrane due to its transparency. The membrane is crosslinked to the hole extent of the polymeric part while in the dry state, the UV light is reflected so that the crosslinking occurs only to a few nanometers depth.

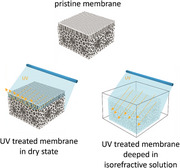

## 1 Introduction

Block copolymers have gained significant interest in recent years as a potential material for the development of membranes due to their unique properties.^[^ ^1^, ^2^ ^]^ The use of block copolymers in membrane applications offers several advantages over traditional materials. One of the most significant advantages is the ability to precisely control the size and shape of the pores within the membrane.^[^ ^3^, ^4^ ^]^ This is achieved by tuning the molecular weight and composition of the polymer blocks, as well as the processing conditions used to form the membrane.^[^ ^5^ ^]^ Another advantage of block copolymer membranes is their high selectivity for specific molecules or ions.^[^ ^6^ ^]^ This selectivity is due to the chemical nature of the polymer blocks, which can be tailored to interact selectively with certain types of molecules or ions. In terms of potential applications, block copolymer membranes have shown promise in a variety of areas, including water purification, gas separation, and drug delivery.^[^ ^7^ ^]^ However, there are still several challenges that need to be addressed before these materials can be widely used in commercial applications. One of the major challenges is the scalability of the membrane formation process, as well as the cost‐effectiveness of producing these materials in large quantities.

Block copolymer membranes have many desirable properties such as high selectivity, tunable pore sizes, and excellent mechanical stability.^[^ ^8^ ^]^ However, they also have some weaknesses that limit their widespread use.^[^ ^9^ ^]^ Block copolymer membranes typically have lower permeability compared to other membrane materials, such as polymeric or ceramic membranes.^[^ ^3^, ^10^ ^]^ This can limit their efficiency and make them less suitable for applications where high flux rates are required.^[^ ^11^ ^]^ A second drawback is the limited thermal stability: Block copolymer membranes may not be able to withstand high temperatures, which can limit their use in certain industrial applications that require high‐temperature environments. Sensitivity to solvents consists of a third drawback: Some block copolymer membranes can be sensitive to solvents, which can cause swelling or degradation of the membrane material. This can limit their use in applications where the membrane is exposed to solvents, or to corrosive or reactive substances. Finally, limited scalability makes it difficult to scale up to industrial production levels, as the fabrication process can be complex and time‐consuming. Crosslinked block copolymer membranes are a type of block copolymer membrane that has been chemically crosslinked to improve mechanical strength, thermal stability, and chemical resistance. Crosslinking involves forming covalent bonds between the polymer chains in the membrane, creating a 3D network that withstands greater stresses and strains. Improved mechanical strength: Crosslinking increases the membrane's mechanical strength, making it more resistant to deformation and damage. Enhanced thermal stability: Crosslinked membranes can withstand higher temperatures without degradation, making them suitable for high‐temperature applications. Better chemical resistance: Crosslinked membranes are less susceptible to chemical and solvent damage, extending their lifespan and broadening their range of applications. Increased selectivity: Crosslinking can affect pore size and distribution, improving selectivity for certain molecules or ions. Additionally, crosslinked membranes are less likely to swell in contact with solvents or other liquids, improving their stability and durability. Despite these advantages, crosslinked block copolymer membranes have some drawbacks. For example, the crosslinking process can make membrane fabrication more complex and time‐consuming, and it can limit the ability to modify membrane properties post‐synthesis. If the crosslinking is too extensive, it can reduce the membrane's permeability, limiting its practical use. Overall, the choice of using a crosslinked or non‐cross‐linked block copolymer membrane depends on the specific application and the desired properties of the membrane.

Membranes are today the standard media for filtration, separation, or purification processes based on the relative size of the materials processed, in either gas or liquid phase separations.^[^ ^12^ ^]^ They offer a wide range of applications in fields of gas separation (separation through solution/diffusion), and liquid separation (ranging from reverse osmosis, nano‐, ultra‐, micro‐filtration up to particle of centimeter sizes) like water purification, wastewater treatment, food industry, the chemical and pharmaceutical as well as in the medical sector. Due to these interdisciplinary fields of applications, there are a few different requirements for membrane performance. Polymer‐based materials are due to their unique and wide‐spread characteristics promising candidates for membrane applications. In this regard, significant progress has been made in the development of polymer and block copolymer membranes having higher selectivity, sharp molecular weight cutoff, and permeability than currently available commercial membrane materials.^[^ ^3^ ^]^

An approach for the preparation of polymeric membrane materials is the use of block copolymers in the SNIPS (a combination of self‐assembly and non‐solvent induced phase separation) process generating integral asymmetric membrane materials.^[^ ^13^ ^]^ The membranes consist of a very thin selective layer with hexagonally ordered pores of uniform size and a random inhomogeneous substructure. Due to these characteristics, block copolymer membranes offer good selectivity, sharp molecular weight cutoff, and permeability compared to commercially available membranes. Based on the SNIPS process, smart polymer‐based materials can create tailor‐made membrane architectures and functionalities for various applications. However, these new membranes lack thermal and chemical stability. They can only be used in aqueous media at low pressure and temperatures below the glass transition temperature and are not widely fabricated on a technical scale.

**Figure 1:**
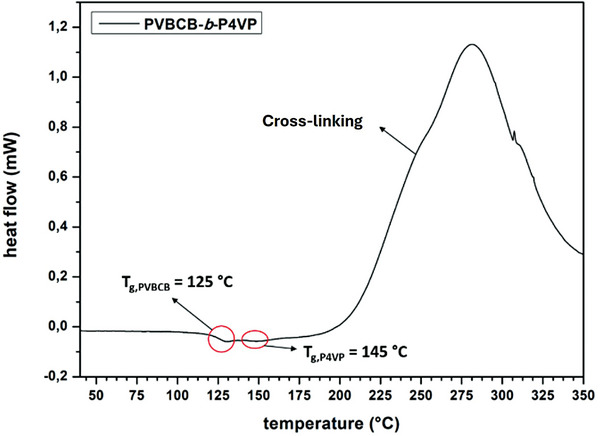
DSC curve of a PVBCB‐*b*‐P4VP block copolymer membrane.

In this work, we propose an easy way to fabricate cross‐linked isoporous integral asymmetric block copolymer‐based membranes. They are prepared by combining the self‐assembly of the block copolymers with the solvent‐non‐solvent induced phase separation process. Afterward, the membranes are cross‐linked by UV irradiation in an isorefractive solvent while maintaining their integral asymmetric structure (**Figure** **1**). UV crosslinking in polymers is a versatile technique that uses ultraviolet light to create chemical bonds (crosslinks) between polymer chains. This process significantly enhances the mechanical strength, durability, and resistance of polymers to various environmental factors. By controlling exposure parameters, such as UV intensity, exposure time, and the choice of photoinitiators, UV crosslinking can be tailored for specific applications, ranging from 3D printing and adhesive bonding to medical device manufacturing.

The initial construction of the membrane provides high selectivity and permeability, while cross‐linking induces high temperature and chemical stability. Cross‐linking allows these membranes to be used for high‐temperature and high‐pressure ultrafiltration, such as wastewater treatment. Additionally, these membranes show high chemical stability in organic solvents, acidic, and basic media, making them suitable for applications in the chemical, pharmaceutical, and medical sectors, like dialysis. The structure is reproducible and can be cross‐linked as a cast stand‐alone film without non‐woven support, expanding potential applications like hosting catalysis reactions. This new cross‐linking strategy can be used as a general method for UV cross‐linking thin and porous films up to 100 µm thickness with a continuous structure.

This work expands the effectiveness of UV treatment on block copolymer membranes to conventional membranes. Specimens of both types are treated with UV radiation in isorefractive solutions and their structural characteristics are subsequently evaluated. The study will explore improved stability, changes in permeability, and alterations in response to external stimuli, with detailed results to be published later. By examining the impact of UV treatment on these membrane types, the research aims to provide insights into the potential benefits and applicability of this approach across various membrane materials.

## 2 Results and Discussion

In earlier studies, we reported on the synthesis of PVBCB‐*b*‐P4VP block copolymers and investigated the membrane formation by the SNIPS process intensively by varying the molecular weight, the polymer concentration of the initial casting solution, and the evaporation time.^[^ ^2^ ^]^ In prior investigations conducted by our research team,^[^ ^14^ ^]^ the primary focus revolved around the application of block copolymers containing styrene derivatives and 4‐vinylpyridine (4VP) for membrane fabrication, with the explicit goal of enhancing membrane thermal characteristics when compared to PS‐b‐P4VP block copolymer membranes. The upcoming generation of integral asymmetric block copolymer membranes is characterized by advancements in stability, favorable water permeability, and responsiveness to external stimuli. To achieve these improvements, integral‐asymmetric membranes were synthesized using block copolymers composed of styrene derivatives and 4‐vinylpyridine, such as PtBS‐*b*‐P4VP (poly(tert‐butylstyrene)‐b‐poly(4vinylpyridine)) and PTMSS‐*b*‐P4VP (poly(trimethlyisilyl‐styrene)‐*b*‐poly(4vinylpyridine)), showcasing highly structured surface features. Notably, the elevated glass transition temperature (Tg) of the matrix‐forming block (PtBS or PTMSS) contributed to bolstered thermal stability and resistance to organic solvents. These membranes demonstrated favorable water permeability and exhibited pH‐responsive behavior. In the corresponding work, we are focusing on the targeted cross‐linking of the isoporous integral asymmetric membranes to generate thermal and chemically stable ultrafiltration membranes. In contrast to former block copolymer membranes^[^ ^9^ ^]^ the PVBCB segments paving the way for stable and sustainable ultrafiltration membranes by UV cross‐linking of the cyclobutene moieties.

### 2.1 Membrane Formation of PVBCB‐*b*‐P4VP Block Co‐Polymers

**Scheme 1:**
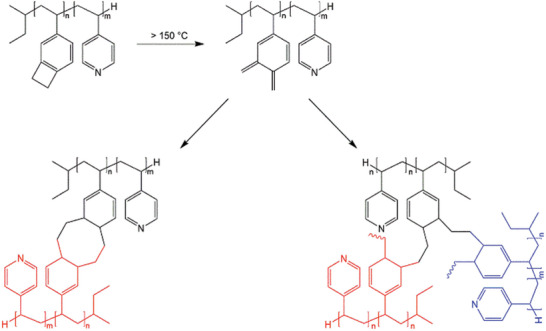
Thermal cross‐linking of the PVBCB moieties. Above 150 °C the benzocyclobutene moieties are transferred into *o*‐xylene units undergoing inter‐and intramolecular reactions resulting in cyclooctadiene (left) and linear polymers (right).

For the fabrication of isoporous integral asymmetric ultrafiltration membranes with hexagonally ordered pores on the surface, block copolymers with defined molecular weights and compositions are basic prerequisites. As already reported in earlier studies^[^ ^2^ ^]^ PVBCB‐*b*‐P4VP block copolymers are synthesized by sequential anionic polymerization in THF at −80 °C with *sec*‐butyllithium (*s*‐BuLi) as initiator. The resulting block copolymers are characterized with respect to their molecular weights and compositions by size exclusion chromatography (SEC) (Figure S1, Supporting Information) and ^1^H‐NMR spectroscopy (Figure S2, Supporting Information). Additionally, the thermal properties of the block copolymers are investigated by differential scanning calorimetry (DSC) measurements (Figure 1). DSC measurements for all block copolymers used in this work reveal one visible glass transition temperature at 125 °C, which can be assigned to the PVBCB block segments. A second glass transition temperature is indicated at ≈145 °C and can be assigned to the P4VP block segments. However, this second glass transition temperature is weakly pronounced due to a very strong exothermic peak starting at 180 °C and showing a maximum at 280 °C. This strong exothermic peak can be assigned to the thermal cross‐linking reaction of the PVBCB block segments. During the heating process, the benzocyclobutene moieties are able to transform into oxylene units. This very reactive species readily undergoes inter‐ and intramolecular cyclo‐reactions like Diels–Alder reaction to produce polycyclic compounds with high stability, such as cyclooctadiene (**Scheme** **1**). The polymerization of o‐quinodinemethane is similar to that of 1,3‐dienes resulting in linear polymers. Ultrafiltration membranes are fabricated from PVBCB‐*b*‐P4VP block copolymers by the SNIPS process. For this purpose, block copolymers are dissolved in a mixture of 45 wt.% THF, 45 wt.% dioxane, and 10 wt.% DMF with polymer concentrations ranging from 20 to 22 wt.%. After solution casting and a certain evaporation time, the membranes are precipitated in a water bath resulting in isoporous integral asymmetric block copolymer membranes with hexagonally ordered pores on the surface.

### 2.2 UV Cross‐Linking of Block Copolymer Membranes

**Figure 2:**
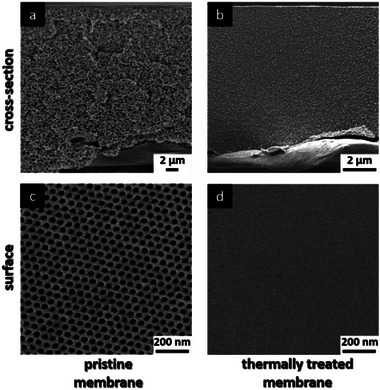
SEM images of the pristine and thermally cross‐linked PVBCB‐*b*‐P4VP block copolymer membrane. a, c) showing the cross‐section and surface of the pristine membrane. b, d) showing the cross‐section and surface of the thermally cross‐linked membrane.

After the successful synthesis and membrane formation process of PVBCB‐*b*‐P4VP block copolymers, the matrix‐building PVBCB block segments offer the opportunity for cross‐linking reactions. As already mentioned the benzocyclobutene moieties can be thermally transferred into *o*‐xylene moieties that rapidly undergo intra‐ and intermolecular cyclo‐reactions and polymerizations resulting in cyclooctadiene segments as well as linear polymers.^[^ ^15^ ^]^ Due to intermolecular reactions, the polymer chains are chemically connected to each other resulting in a 3D cross‐linked network.^[^ ^2^ ^]^ In the case of our membranes, the PVBCB block segments build the matrix offering the possibility of cross‐linking the complete membrane body. However, a thermal cross‐linking of the already fabricated membranes is due to the low glass transition temperatures of PVBCB‐*b*‐P4VP block copolymers not possible. As it can be seen from the DSC measurements the glass transition temperatures of the PVBCB and P4VP block segments are at 125 and 145 °C, respectively. Indeed, the thermal cross‐linking reactions of the PVBCB segments start at temperatures of more than 180 °C, which is above the glass transition temperatures of the block copolymer. Due to the lower glass transition temperature of the polymers in contrast to the cross‐linking temperature the polymer is at 180 °C in the melt state, leading to a high mobility of the chains and resulting in the loss of the characteristic membrane structure. In **Figure** **2** Scanning Electron Microscopy (SEM) images from the cross‐section and the surface of the pristine and the thermally treated PVBCB‐*b*‐P4VP block copolymer membrane are shown. The thermally treated membrane is placed in the oven at 180 °C for 30 min before the SEM measurements. The thermal treatment of the membrane was the only way to visualize the depth of penetration of the UV light in this work. A destructive characterization method for the membrane structure which combined with SEM measurement could reveal to which depth the membrane structure was crosslinked as the rest of it would melt.

**Figure 3:**
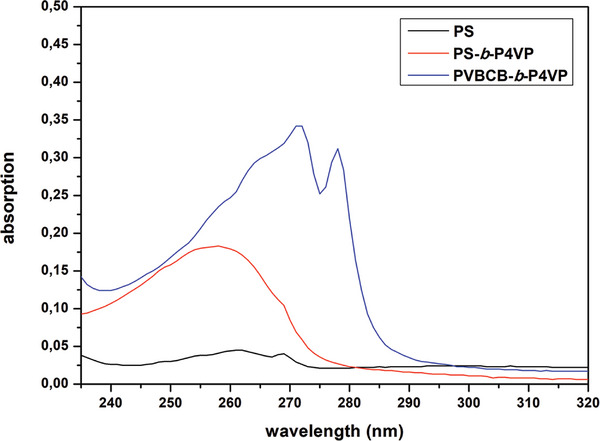
UV/Vis spectra of bulk film from polystyrene (black), PS‐b‐P4VP (red), and PVBCB*‐b*‐P4VP (blue) block copolymer.

As can be seen, the structure of the pristine membrane is destroyed due to the high temperature above the glass transition temperature of the PVBCB*‐b*‐P4VP block copolymer resulting in a dense film (Figure 2b,d). Another opportunity for the external energy input that is needed for the cross‐linking reaction is the use of UV light instead of high temperatures. The UV/Vis spectra of a PVBCB*‐b‐*P4VP block copolymer bulk film as well as the spectra of polystyrene and a PS‐b‐P4VP block copolymer bulk films are shown in **Figure** **3**. The PVBCB‐*b‐*P4VP bulk film absorbs UV light in the range of 240 to 290 nm. When compared to the spectra of polystyrene and PS*‐b*‐P4VP bulk films the cross‐linkable PVBCB block segments are absorbing UV light in the range of 275 to 290 nm. For the UV cross‐linking of the PVBCB‐*b*‐P4VP block copolymer membranes, a xenon UV lamp is used since this type does not heat up the samples and only a UV‐driven reaction takes place. At the beginning of this study, the general concept of the UV cross‐linking strategy is proved by the variation of different parameters. For this purpose, the pristine membrane is placed at a defined distance under the UV lamp and irradiated for a defined time with a defined wavelength and intensity.

First attempts with varying distances between membrane and UV source as well as different intensities indicate that 2 cm and the highest intensity of 300 W reveal the best cross‐linking results. Next to the distance between the membrane and UV source as well as the intensity, also the irradiation time is varied between 4 and 24 h to define the optimum (Figure S5, Supporting Information). To prove the successful cross‐linking the membranes are heated after UV irradiation for 30 min at 180 °C before the cross‐sections and surfaces of the UV irradiated membranes are characterized by SEM measurements (Figure S3, Supporting Information).

**Figure 4:**
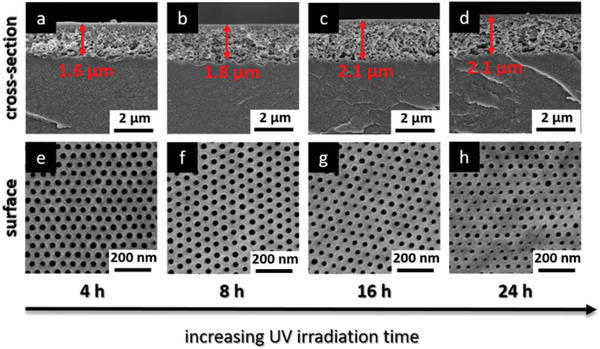
SEM images of the cross‐sections and surfaces of UV irradiated and thermally treated PVBCB‐*b*‐P4VP block copolymer membranes in dependency of the irradiation time. Red arrows indicate the penetration depths of the UV light into the membrane.

The cross‐linked parts of the membrane show an intact structure while the not cross‐linked parts look like a dense film. With this method, it is possible to define the penetration depth of the UV light into the membrane. In **Figure** **4** SEM images of the cross sections and surfaces of the irradiated PVBCB*‐b‐*P4VP block copolymer membranes in dependency of the irradiation time after thermal treatment for 30 min at 180 °C are shown. As can be seen, the cross‐linked parts of the membrane show an intact structure on the surface as well as in the cross‐section due to their thermal stability while the not cross‐linked parts melt due to temperature treatment resulting in dense films. The cross‐linked part also displays the penetration depth of the UV light into the membrane as indicated by the red arrows. By varying the irradiation time between 4 to 24 h the penetration depth of the UV light is increased from 1.6 µm at 4 h to 2.1 µm at 16 h (Figure S5, Supporting Information). By increasing the irradiation time to 24 h no further increase of the cross‐linked part is observed. However, the increase of the irradiation time affects the pore size and pore size distribution resulting in a shrinkage of the pores. Up to 4 h of UV irradiation the hexagonally ordered pores on the surface are unaffected and stay intact while a further increase in the irradiation time leads to smaller pores with a more irregular pore size distribution. All parameters used for the UV cross‐linking as well as penetration depth are listed in **Table** **1**

**Table 1:** Parameters used for the UV cross‐linking of the PVBCB*‐b‐*P4VP membranes, membrane thickness, and penetration depth of UV light are shown in Figure 4.

	Polymer	Distance^a)^ [cm]	Time [h]	Wavelength [nm]	Membrane thickness [µm]	Penetration depth^b)^ [µm]
a	PVBCB‐*b*‐P4VP	2	4	280	24	1.6
b	PVBCB‐*b*‐P4VP	2	8	280	24	1.8
c	PVBCB‐*b*‐P4VP	2	16	280	24	2.1
d	PVBCB‐*b*‐P4VP	2	24	280	24	2.1

^a)^ distance between membrane surface and UV lamp;

^b)^ penetration depth of UV light also indicating the cross‐linked part of the membrane.

**Figure 5:**
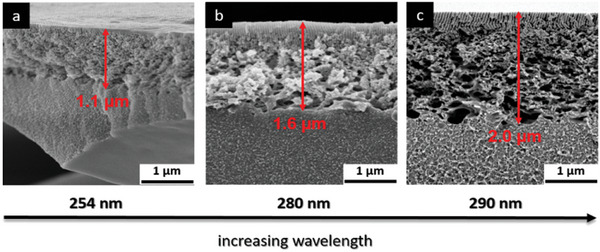
SEM images of the cross‐sections of UV irradiated and thermally treated PVBCB‐*b*‐P4VP block copolymer membranes in dependency of the used wavelength. Red arrows indicate the penetration depths of the UV light into the membrane.

Furthermore, the penetration depths of the UV light in dependency on the wavelength are further investigated. For these experiments, the membranes are irradiated with UV light of different wavelengths for 4 h. The cross‐sections of the irradiated PVBVB‐b‐P4VP block copolymer membranes are characterized by SEM measurements with respect to the penetration depths after thermal treatment at 180 °C for 30 min. The corresponding SEM images in dependency of the wavelengths are shown in **Figure** **5** and the cross‐linked parts are indicated by red arrows. As can be seen, the penetration depth of the UV light increases from 1.1 over 1.6 to 2.0 µm by increasing the wavelength from 254 over 280 to 290 nm. This is expected because UV light with higher wavelengths possesses higher energy and can further penetrate into a material. All parameters used for the UV cross‐linking as well as penetration depth are listed in **Table** **2**. In general, it is not possible to enhance the cross‐linked part of the membrane to more than 2 µm due to the short penetration depth of the UV light into the membrane.

**Table 2:** Parameters used for the UV cross‐linking of the PVBCB*‐b‐*P4VP membranes, membrane thickness, and penetration depth of UV light are shown in Figure 5.

	Polymer	Distance^a)^ [cm]	Time [h]	Wavelength [nm]	Membrane thickness [µm]	Penetration depth^b)^ [µm]
a	PVBCB‐*b*‐P4VP	2	4	254	2.5	1.1
b	PVBCB‐*b*‐P4VP	2	4	280	24	1.6
c	PVBCB‐*b*‐P4VP	2	4	290	24	2.0

^a)^ distance between membrane surface and UV lamp;

^b)^ penetration depth of UV light also indicating the cross‐linked part of the membrane.

#### 2.2.1 UV Cross‐Linking of Block Copolymer Membranes in Solution

**Figure 6:**
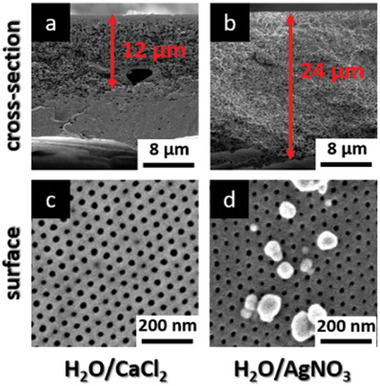
SEM images of the cross‐sections and surfaces of UV irradiated and thermally treated PVBCB‐*b*‐P4VP block copolymer membranes. Red arrows indicate the penetration depths of the UV light into the membrane. a) and c) membrane was soaked for 24 h in an aqueous CaCl_2_ solution before UV irradiation. b) and d) membrane was soaked for 24 h in an aqueous AgNO_3_/PVP solution before UV irradiation.

By varying, the distance between the membrane and the UV source, the intensity and the wavelength as well as the irradiation time it is not possible to cross‐link more than the first 2 µm of the PVBCB‐*b*‐P4VP block copolymer membranes. An opportunity to increase the penetration depths of the UV light resulting in a larger cross‐linked part of the membrane is the filling of the membrane with substances that are able to waveguide UV light. Such wave‐guiding substances can be salts or nanoparticles. For this purpose, the membrane is soaked for 24 h in a saturated aqueous calcium chloride (CaCl_2_) solution before irradiating with UV light. Another approach is the use of silver nanoparticles as waveguides. In this case, we use silver nitrate (AgNO_3_) and poly vinylpyrrolidone (PVP) in water because it is an easy method to generate stabilized silver nanoparticles in situ by irradiation with UV light. Therefore, the membrane is soaked for 24 h in an aqueous AgNO_3_/PVP solution before irradiating with UV light. After UV irradiation, the membranes are again thermally treated at 180 °C for 30 min before the cross‐sections and surfaces are characterized by SEM measurements. The SEM images of the membranes soaked in an aqueous CaCl_2_ and AgNO_3_/PVP solution are shown in **Figure** **6**. As can be seen, it was possible to increase the penetration depth of the UV light and consequently the cross‐linked part of the membrane from 2 to 12 µm by soaking the membrane for 24 h in a saturated aqueous CaCl_2_ solution before irradiation. When using an aqueous AgNO_3_ solution before irradiation the penetration depths are even increased to 24 µm resulting in the complete cross‐linking of the whole membrane body from the top to the bottom as shown in Figure 6b. The in situ generation of silver nanoparticles by UV irradiation is successfully indicated by the particles that can be seen on the membrane surface in Figure 6d. This means that the CaCl_2_ ions as well as the silver nanoparticles act as waveguides and are able to direct the light through the membrane body by scattering the light on the ions or particles. Very important is the use of the right solvent for the soaking solution. It must be a non‐solvent for the membrane or rather for the PVBCB‐*b*‐P4VP block copolymer because in any other case, the membrane starts swelling or getting dissolved leading to a loss of the characteristic structure of the membranes. Therefore, water is used for the PVBCB‐*b*‐P4VP membrane wave‐guiding solutions. All parameters used for the UV cross‐linking as well as the membrane thickness and the penetration depths are listed in **Table** **3**.

**Table 3:** Parameters used for the UV cross‐linking of the PVBCB‐*b*‐P4VP membranes, membrane thickness, and penetration depth of UV light are shown in Figure 6.

Membrane casting solution	Distance^a)^ [cm]	Time [h]	Wavelength [nm]	Membrane thickness [µm]	Penetration depth^b)^ [µm]
H_2_O/CaCl_2_	2	4	280	24	12
H_2_O/AgNO_3_	2	4	290	24	24

^a)^ distance between membrane surface and UV lamp;

^b)^ penetration depth of UV light also indicating the cross‐linked part of the membrane.

Next to the cross‐sections, also the surfaces of the UV cross‐linked membranes were characterized by SEM measurements (Figure 6c,d). In both cases, the isoporous surface structure with hexagonally ordered pores is pronounced but the pores shrink in contrast to the pristine membrane and also the pore size distribution is more irregular than in the case of the original membrane. Additionally, the membrane soaked in an AgNO_3_ solution retains residuals of silver particles in the cross‐section as well as on the surface that cannot be removed by washing steps. These residuals may affect the membrane performance, when thinking about long‐time performance and fouling, indicating that this method should be optimized.

#### 2.2.2 UV Cross‐Linking of Block Copolymer Membranes in Isorefractive Solvents

**Figure 7:**
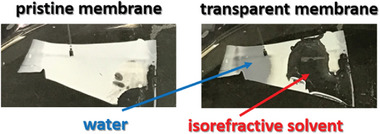
(Left) Photo of the pristine membrane. (Right) Photo of the membrane drop cast with water and an isorefractive solvent.

As reported in the last section it is possible to fill the block copolymer membrane with an aqueous AgNO_3_ solution and to synthesize silver nanoparticles in situ within the membrane by UV irradiation. This also leads to a complete cross‐linking of the whole membrane body due to waveguiding but residuals of the nanoparticles remaining inside the membrane may affect the performance. To circumvent such problems a completely new cross‐linking strategy namely to soak the membrane in a solution that is isorefractive to the membrane as shown in **Figure** **7**. In the case of an isorefractive solvent, this solvent has the same refractive index as the membrane. Therefore, the light cannot differentiate between the solvent and the membrane resulting in a transparent material as shown in Figure 7 on the right side. Normally the membrane is white, which means that the surface can reflect and absorb light leading to an intrinsic boarder for the penetration depths of UV light into the membrane.

**Figure 8:**
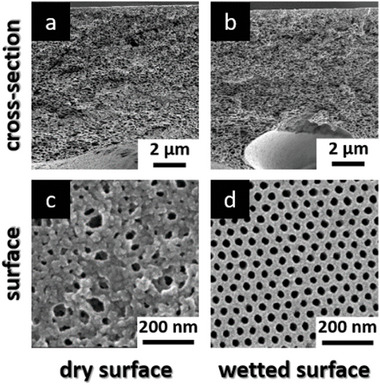
SEM images of the cross‐sections and surfaces of UV irradiated and thermally treated PVBCB‐*b*‐P4VP block copolymer membranes. a, c) membrane was soaked for 24 h in an aqueous ZnBr_2_ solution and the surface was dried before UV irradiation. b, d) membrane was soaked 24 h in an aqueous ZnBr_2_ solution before UV irradiation. The surface was wetted with a thin film of the soaking solution.

In the case of our PVBCB‐*b*‐P4VP block copolymer membranes, this intrinsic boarder is reached at a penetration depth of 2 µm. A solvent that is isorefractive to the PVBCB‐*b*‐P4VP block copolymer membranes must fulfill the following points that a successful cross‐linking is possible: i) the isorefractive solution must have the same refractive index as the membrane material. ii) The solution must be a non‐solvent for the membrane because otherwise, the membrane starts swelling or dissolution and the structure gets destroyed. iii) The solution is not allowed to absorb UV light of the wavelength that is used for the cross‐linking reaction. In our case, the PVBCB‐*b*‐P4VP block copolymer membrane has a refractive index of 1.60, which means that the isorefractive solution also must have a refractive index of 1.60. Solvents possessing such a high refractive index are typically aromatic solvents but due to their electron *π*‐systems, these solvents can interact with UV light. We decided to use a saturated aqueous zinc bromide solution having a refractive index of 1.58. The isorefractive character was proven by placing one droplet of the solution onto the block copolymer membrane resulting in a transparent material as can be seen on the right side of Figure 7. Additionally, aqueous zinc bromide is a non‐solvent for PVBCB‐*b*‐P4VP block copolymers and cannot absorb UV light (Figure S4, Supporting Information), whereby all criteria for cross‐linking with an isorefractive solution are fulfilled (**Figure** **8**).

On the contrary, the membrane covered with a film of the isorefractive solution exhibits a perfect isoporous structure with hexagonally ordered pores on the surface (Figure 7d). It seems that a thin film of the isorefractive solution on the membrane's surface is acting as a protection barrier saving the isoporous surface structure during the cross‐linking process by UV irradiation.

**Figure 9:**
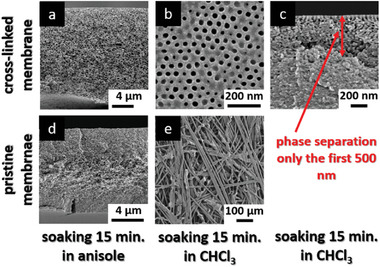
SEM images of the cross‐sections and surfaces of UV irradiated and pristine PVBCB‐*b*‐P4VP block copolymer membranes after chemical treatment. a) The membrane was soaked for 24 h in ZnBr_2_ solution before irradiation and 15 min in anisole after irradiation. b, c) The membrane was soaked 24 h in ZnBr_2_ solution before irradiation and 15 min in chloroform after irradiation. d) Pristine not‐irradiated membrane was soaked for 15 min in anisole before SEM measurements. e) Pristine not‐irradiated membrane was soaked for 15 min in chloroform before SEM measurements.

Next to the thermal stability, the cross‐linked membranes are also tested for chemical stability focusing on organic solvents. For this purpose, the pristine and the cross‐linked membranes were soaked in chloroform for 15 min, respectively. Afterward the cross‐sections and surfaces of the pristine as well as the cross‐linked membranes were characterized by SEM measurements (**Figure** **9**). As can be seen, the isoporous surface structure of the cross‐linked membrane remained unaffected after chloroform treatment (Figure 9b) while the pristine membrane is completely dissolved in chloroform and only the fibers of the non‐woven support are visible (Figure 9e). However, the cross‐section of the cross‐linked membrane (Figure 9c) shows that the integral asymmetric structure of the membrane is intact only for the first 500 nm while the rest of the membrane is a dense film. This phenomenon can be explained by the membrane formation via the SNIPS process. During this membrane formation process, micelles are formed that self‐assemble on top of the casting solution resulting in open cylindrical pores on the membrane's surface. However, these block copolymer self‐assembly only takes place on top of the solution generating the membrane's surface and not throughout the whole membrane body. The cross‐linking reaction only takes place in the PVBCB block segments due to the benzocyclobutene moieties but not in the P4VP segments. This means that chloroform is not able to dissolve the cross‐linked PVBCB segments while it is able to dissolve the P4VP block segments. In the phase‐segregated parts of the membrane, in our case the first 500 nm, the structure is not affected due to the phase separation of the PVBCB and P4VP block segments during the SNIPS process. In the rest of the membrane body, no phase separation between both block segments took place leading to the dissolution of the P4VP moieties with the loss of the structure and resulting in a dense film, consequently.

The chemical stability of the cross‐linked membrane is further investigated by soaking both, the pristine and the cross‐linked membrane for 15 min in anisole. Anisole is a good solvent for PVBCB but a non‐solvent for P4VP. As can be seen in Figure 9a the integral asymmetric structure of the cross‐linked membrane remained unaffected through the whole membrane body while the pristine membrane was swollen by anisole resulting in a dense film. To summarize, it is possible to cross‐link the PVBCB‐*b*‐P4VP block copolymer membranes throughout the whole membrane thickness by using an isorefractive solution yielding thermal and chemically stable ultrafiltration membranes.

### 2.3 UV Cross‐Linking of Commercial Ultrafiltration Membranes

In the last step of our study, the new cross‐linking strategy is transferred from block copolymer membranes to commercial ultrafiltration membranes. For this purpose, commercial PVDF and Versapor 1.200 (consisting of PAN‐*co*‐PVC statistical copolymer) membranes from PALL Life Science were used. PVDF and Versapor 1.200 have refractive indices of 1.42 and 1.52, respectively. DMSO and a saturated solution of zinc bromide in butanol were used as isorefractive solvents for the PVDF and Versapor 1.200 membranes. Up to a temperature of 50 °C DMSO is a non‐solvent for PVDF while zinc bromide solutions are a solvent for PAN but a non‐solvent for PVC. In contrast to PVBCB‐*b*‐P4VP block copolymers, PVDF and PAN‐*co*‐PVC statistical copolymers do not carry any UV active groups that can be used for cross‐linking reactions. Therefore the UV initiators benzophenone and Irgacure 2959 as well as the bifunctional monomer *N*,*N′*‐Methylbis‐acrylamide are added to the isorefractive solvents. Benzophenone is a Norrish‐Type II photoinitiator that can generate radicals in the polymer backbone by abstracting a proton while Irgacure 2959 is a Norrish‐Type I photoinitiator that can start radical polymerizations due to UV irradiation. The bifunctional monomer *N*,*N′*‐Methylbis‐acrylamide acts as a photo cross‐linker that can be polymerized during UV irradiation. It is important that the photoinitiators and the cross‐linker can be dissolved in the isorefractive solvents. To cross‐link the commercial membranes, the materials were placed at a 2 cm distance to the UV source and irradiated for 4 h with unfiltered UV light covering wavelength in the range of 280 to 320 nm. Before UV irradiation, the membranes were soaked for 24 h in the isorefractive cross‐linking solutions. All parameters used for the cross‐linking of PVBCB‐*b*‐P4VP block copolymer, PVDF, and Versapor 1.200 membranes are summarized in **Table** **4**.

**Table 4:** Parameters used for the UV cross‐linking of PVBCB‐*b*‐P4VP membranes as well as commercials Versapor 1.200 and PVDF membrane.

Membrane	Isorefractive casting solution	Cross‐linking agents	n_membrane_ ^a)^	n_solution_ ^b)^
PVBCB‐*b*‐P4VP	H_2_O/ZnBr_2_	–	1.60	1.58
Versapor 1.200	BuOH/ZnBr_2_	Benzophenone Irgacure 2959 *N*,*N′*‐Methylbisacrylamide	1.52	1.50
PVDF	DMSO	Benzophenone Irgacure 2959 *N*,*N′*‐Methylbisacrylamide	1.43	1.48

^a)^ refractive index of the ultrafiltration membrane;

^b)^ refractive index of the isorefractive casting solution used for cross‐linking of the membrane.

**Figure 10:**
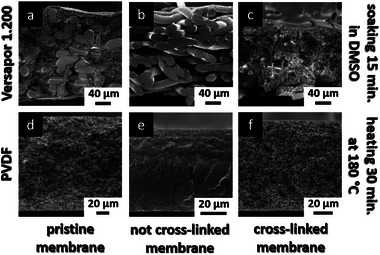
SEM images of the cross‐sections of UV irradiated and pristine commercial ultrafiltration membranes after chemical or thermal treatment. a) Pristine commercial membrane Versapor 1.200 without chemical or thermal treatment. b) Pristine not‐irradiated membrane Versapor 1.200 was soaked 15 min in DMSO before SEM measurements c) Membrane Versapor 1.200 was soaked 24 h in BuOH/ZnBr_2_ solution before irradiation and 15 min in DMSO after irradiation. d) Pristine commercial PVDF membrane without chemical or thermal treatment. e) Pristine not‐irradiated PVDF membrane was thermally treated before SEM measurements f) PVDF membrane was soaked in 24 h isorefractive solution before irradiation and thermally treated after irradiation.

After UV irradiation, the membranes are washed with DMSO or butanol and water. To prove the concept of the UV cross‐linking by using isorefractive solvents the pristine and the cross‐linked PVDF membrane are thermally treated at 180 °C for 30 min before cross‐sections were characterized by SEM measurements. In the case of Versapor 1.200 the pristine and the cross‐linked membrane were soaked for 15 min in DMSO before cross‐sections were characterized by SEM measurements. All SEM images are shown in **Figure** **10**. As can be seen, the cross‐linked and thermally treated PVDF membrane (Figure 10f) shows a porous membrane structure that is identical to the porous structure of the pristine untreated PVDF membrane (Figure 10d). In contrast to the cross‐linked membrane the pristine thermally treated membrane does not show any porous structure and is only a dense film indicating the successful cross‐linking of the UV‐irradiated membrane. In the case of Versapor 1.200 the cross‐linked membrane also shows a porous membrane structure after chemical treatment in DMSO (Figure 10c) even if the structure is not identical to the pristine membrane (Figure 10a). This can be explained by the use isorefractive solution. As already mentioned, zinc bromide solutions are good solvents for PAN for which reason the membrane starts swelling during the cross‐linking process and the original structure is getting destroyed.

When compared to the pristine membrane after chemical treatment the membrane is completely dissolved in DMSO and only the fibers of the support are visible. These findings indicate the successful cross‐linking of the Versapor membrane. The used isorefractive solvents as well as the concentrations of the photoinitiators and cross‐linkers have to be optimized. In general, the first proof of concept for using isorefractive solutions with photoinitiators and UV cross‐linkers to generate thermal and chemically stable ultrafiltration membranes by using UV cross‐linking is considered successful. With this new strategy, it is possible to fabricate membranes that are cross‐linked throughout the whole membrane thickness resulting in thermal and chemical stable ultrafiltration membranes. The cross‐linking strategy using isorefractive solvents can be easily transferred to every porous material and may pave the way for more sustainable porous materials and membranes.

## 3 Conclusion

The present work is a follow‐up study of a previous work where we reported on the membrane formation of new PVBCB‐*b*‐P4VP block copolymer membranes by the SNIPS process.^[^ ^2^ ^]^

Within this study, we focused on the cross‐linking of the PVBCB‐*b*‐P4VP block copolymer membranes. The benzocyclobutene moieties in the PVBCB block segments reveal the opportunity for undergoing intermolecular cross‐linking reactions when irradiated with UV light. The penetration depths of the UV light indicated by the cross‐linked part of the membrane in dependency on the distance between the membrane and the UV source, the irradiation time, the intensity, and the used wavelength were investigated. It was shown that the penetration depths of the UV light had an intrinsic boarder and it was not possible to cross‐link more than 2 µm of the membrane. In general, the penetration depth was determined by SEM measurements. Therefore, the UV irradiated membranes were thermally treated at 180 °C for 30 min yielding dense films at the not cross‐linked parts and intact asymmetric structures in the cross‐linked parts of the membrane.

In the next step, it was shown that the penetration depth of the UV light could be dramatically increased to 12 and 24 µm when soaking the membranes in solutions that are able to waveguide the light. As wave‐guiding solutions aqueous calcium chloride and silver nanoparticle solutions were used. The ions and nanoparticles are able to direct the light yielding higher penetration depths of the UV light. Furthermore, we introduced a completely new and easy strategy for fabricating block copolymer membranes that are cross‐linked throughout the whole membrane thickness. It was shown that the block copolymer membranes could be completely cross‐linked from the top to the bottom when soaking in an isorefractive solution before UV irradiation. For the PVBCB‐*b*‐P4VP block copolymer membranes, a saturated aqueous zinc bromide solution was used having the same refractive index as the block copolymer resulting in a transparent membrane. Additionally, the chemical and thermal stability of these completely cross‐linked block copolymer membranes was shown. Finally, the cross‐linking strategy based on isorefractive solvents was successfully transferred to commercial PVDF and PAN‐*co*‐PVC polymer membranes. Due to the lack of UV active groups in these polymeric membranes photoinitiators of Norrish‐Type I and II as well as a bifunctional UV active cross‐linker were added to the isorefractive solvents. The UV irradiation resulted in completely cross‐linked PVDF and PAN‐*co*‐PVC polymer membranes showing thermal and chemical stability. In general, the new cross‐linking strategy using isorefractive solvents can be easily transferred to every porous material and may pave the way for more stable and sustainable porous materials and membranes.

## 4 Experimental Section

### Reagents

All solvents and reagents were purchased from Alfa Aesar, Sigma–Aldrich, Fisher Scientific, and ABCR, and used as received unless otherwise stated. Tetrahydrofuran (THF) was distilled from sodium/benzophenone under reduced pressure (cryo‐transfer) prior to the addition of 1,1 diphenylethylene and *n*‐butyllithium (*n*‐BuLi) followed by a second cryo‐transfer. Vinylbenzocyclobutene (VBCB) (Sigma–Aldrich) was dried over calcium hydride and distilled twice from di‐*n*‐butylmagnesium by cryo‐transfer. 4‐Vinylpyridine (4VP) (Sigma–Aldrich) was distilled first from calcium hydride and afterward cryo‐transferred from ethyl aluminum dichloride prior to use in polymerization. All polymerizations were carried out in a Schlenck line apparatus using a high vacuum (10^−7^–10^−8^ mbar) and Argon (Argon 7.0, Linde AG).

### Instrumentation

NMR spectra were recorded on a Bruker Ascend 500 NMR spectrometer working at 500 MHz (^1^H‐NMR) using CDCl_3_ and DMF‐d7 as solvents at room temperature. NMR chemical shifts were referenced relative to tetramethylsilane (TMS). Standard SEC was performed with a system composed of a VWR‐Hitachi 2130 pump and a Shodex RI‐101 refractive index detector at 30 °C, CHCl_3_ as the mobile phase (flow rate 1 mL min 1) on a GRAM column set from PSS (GRAM precolumn (dimension 8·50 mm), GRAM column (porosity 3000 A, dimension 8·300 mm, particle size 10 µm) and GRAM column (porosity1000 A, dimension 8·300 mm, particle size 10 µm)). Calibration was carried out using PS standards (from Polymer Standard Service, Mainz). For data acquisition and evaluation of the measurements, PSS WinGPC UniChrom 8.2 was used. SEM measurements were performed on a LEO Gemini 1550 VP (Zeiss, Oberkochen, Germany) at an operating voltage of 3−5 kV using a secondary electron detector. Prior to SEM measurements, the polymer samples were coated with 2 nm platinum using a sputter coater. Cross‐sections of the membranes were prepared while dipping the membranes in iso‐propanol, freezing them in liquid nitrogen, and breaking them. Average pore size values were determined using the software analysis (Olympus Soft Imaging Solutions GmbH, Münster, Germany) on the basis of the SEM results. For determination of the thermal properties of the polymers differential scanning calorimetry (DSC) was performed with a Mettler Toledo DSC‐1 in a temperature range from −100 to 150 °C with a heating rate of 10 K min^−1^. UV‐Lamp (Model: MAX‐303 – ASAHI SPECTRA, 300 W xenon light source monochromatic light with Filters: 250 – 1050 nm ‐it depends on the mirror module).

### Anionic Polymerization

Anionic Polymerization of Vinylbenzocyclubutene and 4‐vinylpyridine exemplary synthesis of a poly(vinylbenzocyclobutene‐*block*‐4‐vinylpyridine) featuring a molar mass of 109 kg mol^−1^ (PVBCB_79_‐*b*‐P4VP_21_^109k^). In a 250 mL glass reactor equipped with a stirring bar, subsequently purified THF was distilled and titrated under argon by a small amount of *s*‐BuLi until a vivid yellow color was observed. Upon the disappearance of the color the reactor was brought to −80 °C and 2.213 g (0.023 mol, 1045 eq.) of purified 4‐vinylbenzocyclobutene was added to the reactor. The polymerization was initiated by the quick addition of 0.08 mL *s*‐BuLi (0.000022 mol, 1 eq., 0.28 m solution in cyclohexane) with a syringe. The solution was stirred for 1 h at −80 °C to ensure complete conversion of 4‐vinylbenzocyclobutenes before an aliquot of the solution was taken from the reactor for characterization of the PVBCB segment and terminated with methanol. Afterward, 0.539 g (0.0056 mmol, 255 eq.) purified 4‐vinylpyridne was added to the solution and stirred for an additional 24 h. After adding a small amount of degassed methanol, the polymer was precipitated in a tenfold excess of hexane. The polymer was collected by filtration, washed with hexane, and dried in a vacuum (yield: 2.75 g, 96%).

GPC (vs PS): PVBCB: Mn = 85 900 g mol^−1^; Mw = 88 500 g mol^−1^; Đ = 1.03

PVBCB‐b‐P4VP: Mn = 85 600 g mol^−1^; Mw = 89 700 g mol^−1^; Đ = 1.05

^1^H‐NMR (500 MHz, 300 K, CDCl3, δ in ppm): 7.18–6.91 (br, H4/5), 6.78–6.33 (br, H3), 3.60 (br, H8), 2.24–0.83 (alkyl).

### Membrane Fabrication of PVBCB‐b‐P4VP BCP's by SNIPS process

PVBCB_79_‐*b*‐P4VP_21_^108^ was dissolved in a mixture of dimethylformamide (DMF), dioxane (DIOX), and tetrahydro‐furane (THF) to provide a viscus but clear casting solution. The composition of the casting solution was 20 wt.% PVBCB‐*b‐*P4VP, 36 wt.% THF, 36 wt.% DIOX, and 8 wt.% DMF. The casting solution was spread out using a homemade casting machine using a doctor blade with a gap height adjusted to 200 µm on a polyester nonwoven support. After 10 s, the film was immersed in a water bath. Drying of the membrane follows at 60 °C under vacuum.

## Conflict of Interest

The authors declare no conflict of interest.

## Supporting information

Supporting Information

## Data Availability

The data that support the findings of this study are available from the corresponding author upon reasonable request.
