# Supplementary material for: Enhanced UV Penetration and Cross‐Linking of Isoporous Block Copolymer and Commercial Ultrafiltration Membranes using Isorefractive Solvent
Source: Adv Sci (Weinh). 2024 Jul 1;11(33):2403288. doi: 10.1002/advs.202403288 (PMC11434031; doi:10.1002/advs.202403288)
Supplement: Supplementary file 1 — Supporting Information [file ADVS-11-2403288-s001.docx]

**sSupporting Information**

**Enhanced UV Penetration and Cross-linking of Isoporous Block Copolymer and Commercial Ultrafiltration Membranes using Isorefractive Solvent**

Michael Appold, Sofia Rangou*, Sarah Glass, Brigitte Lademann, Volkan Filiz*

Institute of Membrane Research, Helmholtz-Zentrum Hereon, Max-Planck-Str.1, 21502 Geesthacht, Germany.

Corresponding authors: Sofia Rangou – e-mail: sofia.rangou@hereon.de, Volkan Filiz - e-mail:, volkan.filiz@hereon.de

***Polymer synthesis****:* The polymers of this work were synthesized via sequential anionic polymerization. The nomenclature followed is PVBCB_79_*-b-*P4VP_21_^108k^ where the PVBCB is poly(vinylbenzocyclobutene), P4VP abbreviation for poly(4-vinylpyridine), subscripts the weight percentage of each block in the polymer and the Number following is attributed to the total molecular weight in kg/mol. All monomers and solvents were prior purified to reach the standards for anionic polymerization. More specific the monomers vinylbenzocyclobutane (VBCB, Sigma-Aldrich, Schnelldorf, 97%) was twice distilled from di-n-butylmagnesium (Sigma-Aldrich, Schnelldorf, 1.0 M solution in heptane) under high vacuum and 4-Vinylpyridine (4VP) (Sigma-Aldrich, Munich, Germany) was distilled once from calcium hydride and twice from ethyl aluminum dichloride (Sigma-Aldrich, Schnelldorf, 1.0 M in hexanes). The polymerization procedure is conducted polymerization in a Schlenk line apparatus using high vacuum (10^-7^-10^-8^mbar) and Argon supply (Argon 7.0, Linde AG, Pullach, Germany) as follows: A 250ml glass reactor was connected on the vacuum line, evacuated to attain high vacuum. Subsequently purified THF was distilled into the reactor and titrated under argon at -80 °C, by a small amount of *sec*- butyllithium (s-BuLi) (Sigma Aldrich, Schnelldorf, Germany, 1.4 M solution in cyclohexane), until a vivid yellow color was observed. Upon the disappearance of the color the reactor was brought again at -80 °C and the first purified monomer 4- vinylbenzocyclobutene (4-VBCB 2.213 g, 0.023 mol) was inserted via a syringe in the reactor, followed by the initiator *s*-BuLi (0.28 M in cyclohexane, 0,08 mL, 0.000022 mol). In the polymerization solution immediately developed a bright orange color indicating the formation of propagating anion of 4-VBCB and the reaction left to complete for 1h at -80 °C. After the reaction was completed an aliquot was withdrawn and the second purified monomer 4-vinylpyridine (4-VP, 0,539g 0,0056 mol) was inserted in the polymerization reactor. At this point, the solution color changed rapidly in light yellow-green indicating the propagation of the 4VP block. The polymerization completed overnight and on the following day terminated with vacuum-degassed methanol (0.5 mL) The diblock copolymer was recovered by precipitation in hexane and dried under vacuum at 50 °C for 48 h. The yield is 96% (2.75 g). The molecular characteristics of the diblock copolymer derived from the measurements in GPC using as solvent chloroform and PS standards and ^1^H-NMR in CDCl_3_. The total molecular weight of the polymer is calculated 108kg/mol and PVBCB block is 79 wt.% and P4VP 21 wt.%.

***Molecular Characterization***

Figure S1: Chromatograms of the PVBCB precursor homopolymer (green line) and the final diblock copolymer PVBCB-b-P4VP (blue line) in CHCl_3_

**Gel Permeation chromatography (GPC)**

The molecular weights of the polystyrene-precursor and molecular weight distribution of the block copolymer were determined by gel permeation chromatography calibrated with PS standards. The measurements were performed at 30 °C in chloroform using PSS GRAM columns [GRAM precolumn (dimension 8·50 mm), GRAM column (porosity 3000 A, dimension 8·300 mm, particle size 10 μm) and GRAM column (porosity 1000 A, dimension 8·300 mm, particle size 10 μm)], at a flow rate of 1.0 mL min^-1^ (VWR-Hitachi 2130 pump). A Shodex RI-101 refractive index detector with a polystyrene calibration was used. P4VP, due to its polar nature, has been reported to occasionally exhibit interactions with the stationary phase. Therefore, interactions of these solutes are exhibited asadsorption (retardation in retention time) led to misleading results concerning the actual molecular weight of the second block. For this reason, GPC results from the precursor PVBCB block (Figure S1 green line) were evaluated with the weight average results for each block derived from ^1^H-NMR to reveal the final molecular weight of the block polymer. For the above-mentioned reason the Figure S1 chromatograms are representative for the successful synthesis of a monodispersed homopolymer ((Figure S1 green line) and diblock copolymer polymer (Figure S1 blue line) but in the case of the diblock copolymer not representative total molecular weight.

**Proton Nuclear Magnetic Resonance Spectroscopy (^1^H-NMR)**

The synthesized compounds were analyzed by proton nuclear magnetic resonance spectroscopy (NMR). ^1^H-NMR measurements were performed on a Bruker Ascend 500 NMR spectrometer (500 MHz) using CDCl_3_ and DMF-*d7* as solvent, at room temperature.


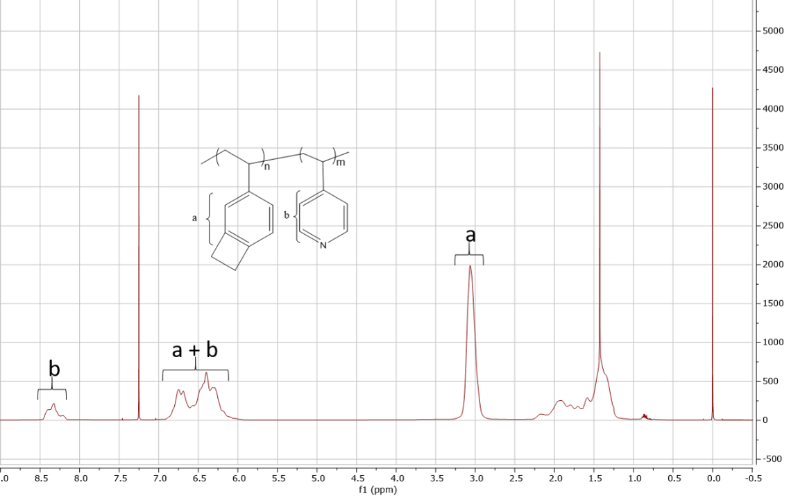


Figure S2: ^1^H-NMR spectrum of the PVBCB_79_-b-P4VP_21_^108k^ block copolymer

P4VP amount was calculated at 21 wt %, P4VCB amount 79 wt%.

**
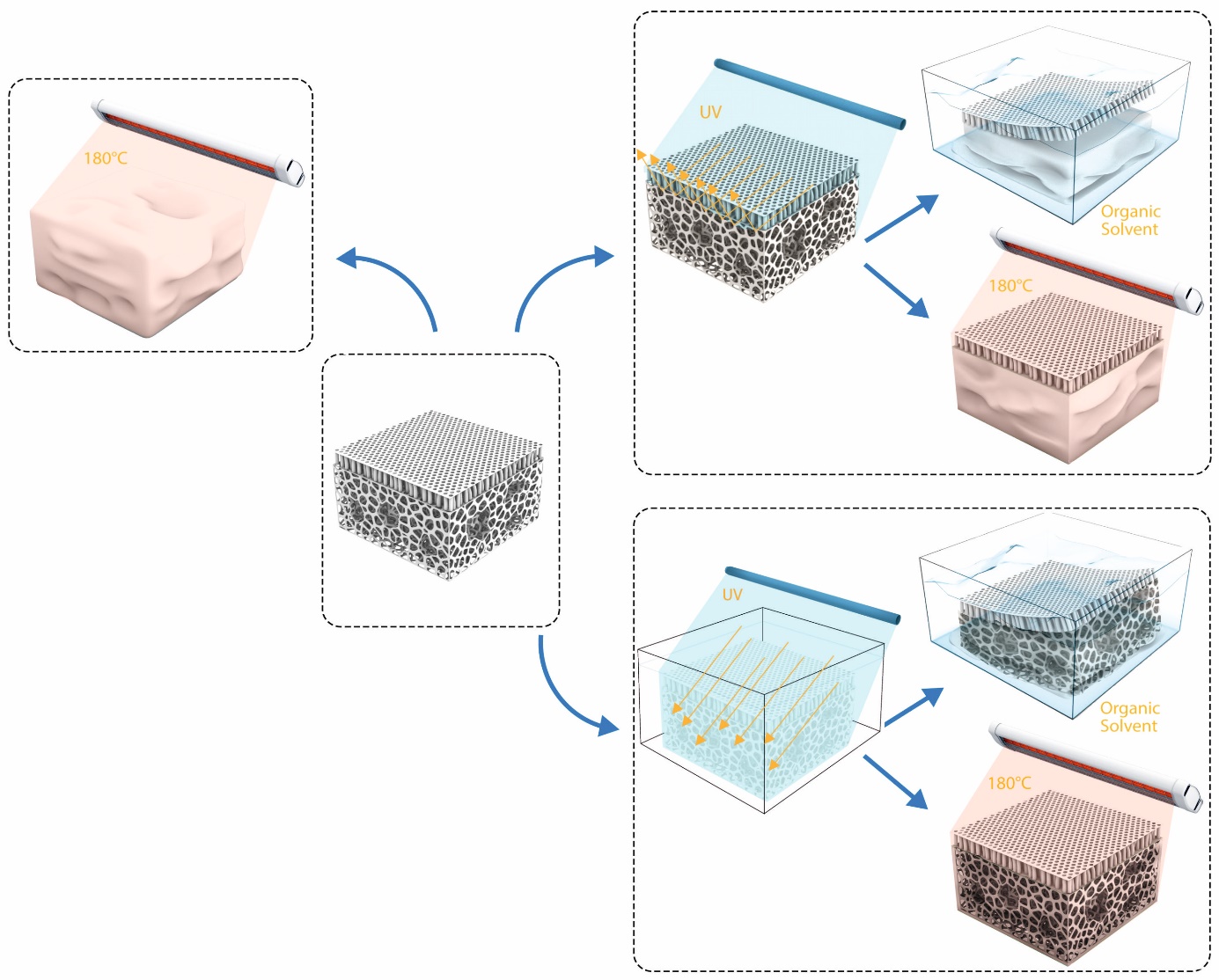
**

D

A

B

C

*Figure S3:* Different attempts that were used during this work in order to achieve the crosslinked polymeric membranes. In the middle a schematic representation of the pristine polymeric membrane is shown (A). In the left side the section is thermally treated and the morphology of the membrane is destroyed (B). In the upper right side the membrane section is exposed to UV light. The UV light is penetrating only the upper part of the membrane. In this case further treatment either chemical or thermal leads to the disappearance of the isoporous structure in the parts which are not crosslinked (C). On the contrary when the section is dipped into isorefractive solvent the entire membrane body is penetrated with UVlight and thus probably all vinylcyclobutane units are cross linked. Further treatment with either temperature or solvents showed that the membrane is remaining unaffected (D).

*Figure S4:* UV-vis adsorption spectra of the ZnBr_2_ aqueous solution (iserefractive solvent)

**Pristine membrane - variation time**


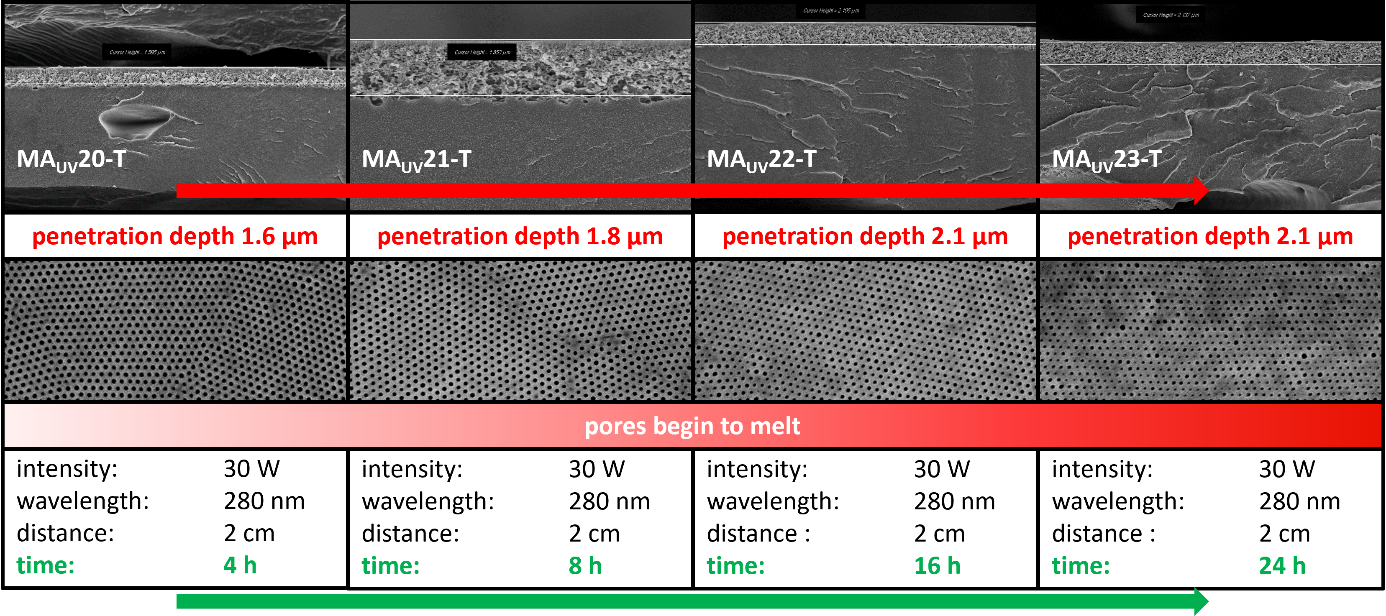


**Additive ZnBr_2_ - variation time-dried samples**


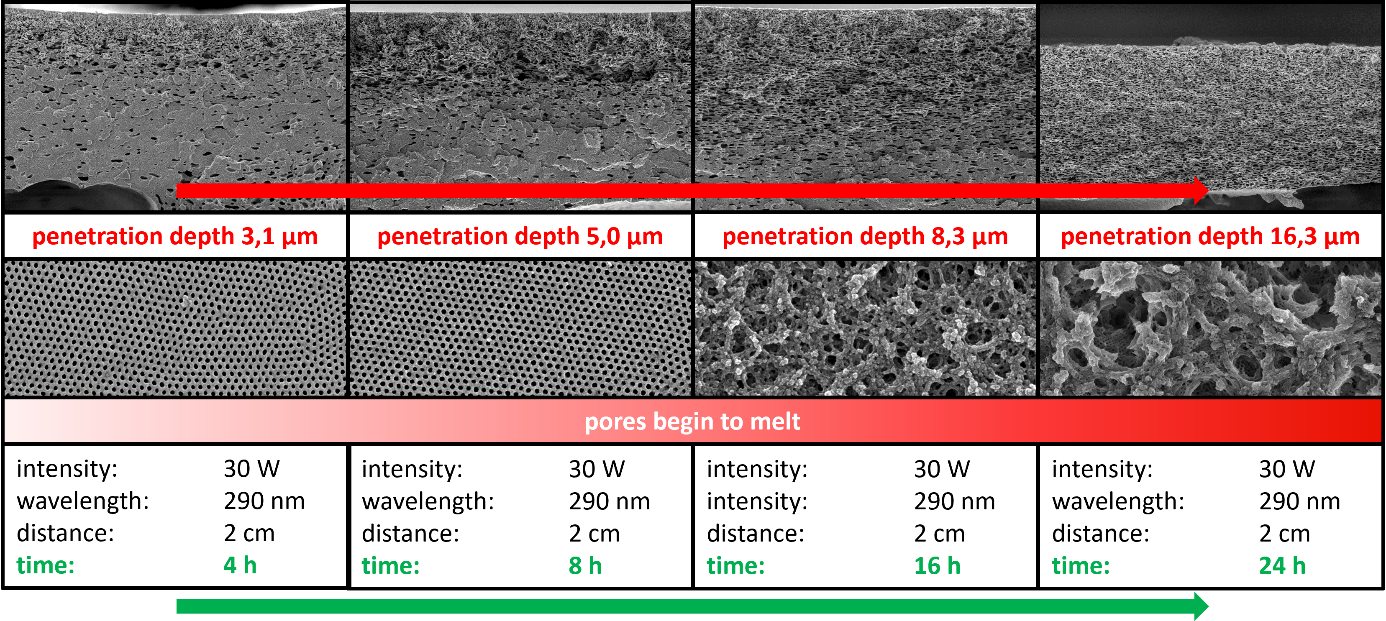


Figure S5: In the upper set of micrographs is showing how the increase of time of UV-light exposure is affecting the extent of the crosslinking of the pristine membrane. After the crosslinking By UV light the sample was treated in high temperature so the non-crosslinked part would melt, and a difference can be seen in the cross-section of the SEM sample. In the lower series the samples were dipped in ZnBr_2_ isorefractive solvent.

**Additive ZnBr_2_ - variation time -high intensity -dried samples**

*
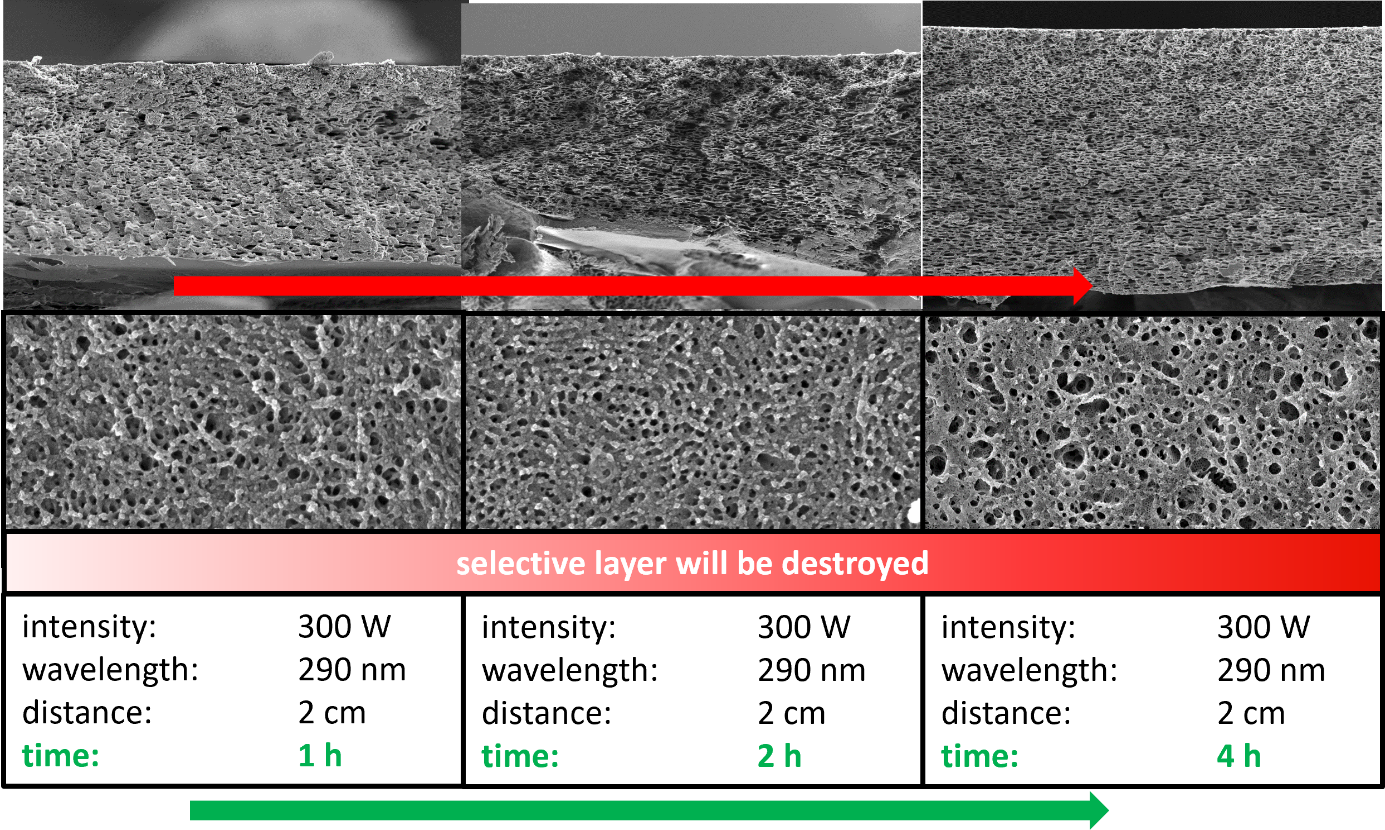
*

*Figure S6*: Increase of the intensity significantly reduces the time of UV treatment of the sample. The crosslinking in this case was also throughout the body of the membrane.


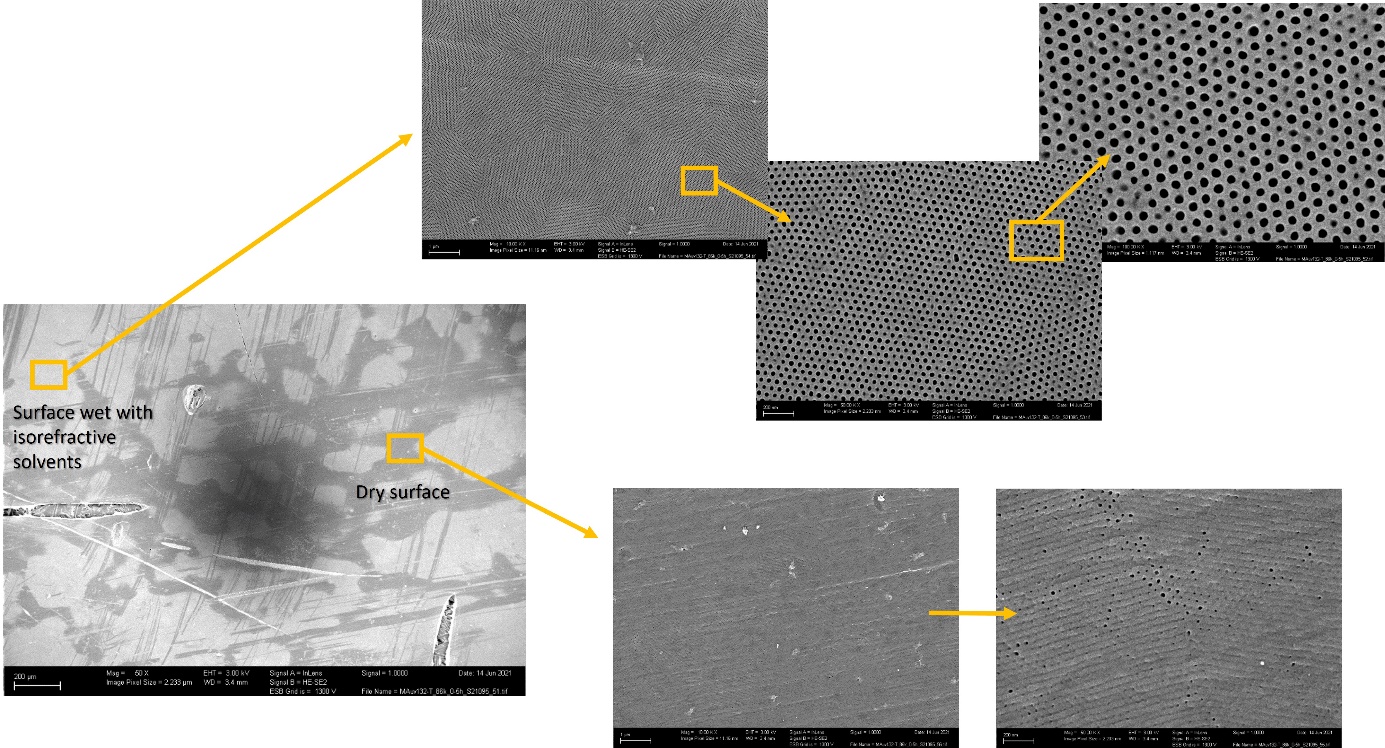


*Figure S7*: Schematic illustration of the isorefractive solvent’s role in maintaining membrane morphology. The highly concentrated salt solution acts as a support for the polymeric branches, filling the gaps within the porous structure and the volume between polymeric chains. This support mechanism helps preserve the membrane’s overall structure and morphology.
